# Cellular Diversity and Differential Subcellular Localization of the G-Protein G_αo_ Subunit in the Mouse Cerebellum

**DOI:** 10.3389/fnana.2021.686279

**Published:** 2021-06-25

**Authors:** Alberto Roldán-Sastre, Carolina Aguado, Alejandro Martín-Belmonte, Rocío Alfaro-Ruiz, Ana Esther Moreno-Martínez, Rafael Luján

**Affiliations:** Synaptic Structure Laboratory, Instituto de Investigación en Discapacidades Neurológicas (IDINE), Department of Ciencias Médicas, Facultad de Medicina, Universidad de Castilla-La Mancha, Albacete, Spain

**Keywords:** G protein, GPCRs, cerebellum, GABA_*B*_ receptors, immunohistochemistry, electron microscopy, Purkinje cell

## Abstract

Heterotrimeric guanine nucleotide-binding proteins (G proteins) transduce signals from G protein-coupled receptors (GPCRs) to effector ion channels and enzymes G_αo_, a member of the pertussis toxin-sensitive G_*i/o*_ family, is widely expressed in the brain, although its role within a neuronal context remains largely unknown. Using immunohistochemical and quantitative immunoelectron microscopy techniques, we have investigated the expression, cellular and subcellular localization of G_αo_ in the cerebellar cortex. Histoblot revealed that G_αo_ is expressed in many brain regions, including the cerebellum. At the cellular level, G_αo_ protein was distributed in Purkinje cells, basket cells, stellate cells, granule cells and Golgi cells. At the subcellular level, pre-embedding immunoelectron microscopy revealed mainly a postsynaptic localization of G_αo_ along the extrasynaptic plasma membrane of Purkinje cell dendritic shafts and spines, and dendrites of basket, stellate and granule cells. To a lesser extent, immunolabeling for G_αo_ was localized in different types of axon terminals establishing excitatory synapses. Moreover, post-embedding immunoelectron microscopy revealed the synaptic localization of G_αo_ on PSDs of glutamatergic synapses between Purkinje cell spines and parallel fiber terminals and its co-localization with GABA_*B1*_ in the same spines. Quantitative analysis of G_αo_ immunoparticles revealed they preferentially localized on the cytoplasmic face of the plasma membrane. Furthermore, the analysis revealed a high concentration of G_αo_ around excitatory synapses on Purkinje cell dendritic spines, but a uniform distribution in granule cell dendrites. These molecular-anatomical findings suggest that G_αo_ is a major signal transducer of specific GPCRs in different neuronal populations in the cerebellum.

## Introduction

The interplay among neurons of the cerebellar cortex is key to achieving different functions, including fine motor control, maintenance of balance and posture, perception, memory and cognition (Ito, 2001, 2006). The cerebellar cortex is a trilaminar structure formed by the molecular layer, the Purkinje cell layer, and the granule cell layer. Purkinje cells (PCs), the only output neurons of the cerebellar cortex, extend their dendrites through the molecular layer where they receive inputs from climbing fibers. They also receive inputs from parallel fibers originating in granule cells (GCs), which integrate sensory information arriving through the mossy fibers to modulate the activity of PCs (Ito, 2001, 2006). In addition to ion channels activation that cause the firing of neurons, the function of cerebellar cells also depends on the signaling through G protein-coupled receptors (GPCRs) transducing stimuli across the plasma membrane (Oldham and Hamm, 2008; Weis and Kobilka, 2018).

In the brain, GPCRs contribute to the regulation of neurotransmission and neuronal excitability (Wettschureck and Offermanns, 2005; Oldham and Hamm, 2008). The activation of GPCRs induces a conformational change that modifies the function of associated intracellular GTP binding proteins (G-proteins), consisting of three subunits, α, β, and γ (Oldham and Hamm, 2008) that transduce extracellular signals from GPCRs to downstream effector molecules such as enzymes and ion channels (Oldham and Hamm, 2008). This process requires an exchange of GDP for GTP on the coupled G-protein α subunit, leading to dissociation of G_βγ_ subunits (Oldham and Hamm, 2008). The dysfunction in GPCR signaling, frequently caused by abnormal activation or overexpression of their associated G proteins, can lead to brain diseases including depression, Parkinson’s disease, Alzheimer’s disease, Huntington’s disease or multiple sclerosis (Borroto-Escuela et al., 2017; Azam et al., 2020). Thus, targeting G proteins instead of GPCRs provides alternative molecular targets in drug discovery for combating diseases that affect different organs including the brain (Li et al., 2020).

The functional diversity of G proteins is paralleled to the molecular diversity of G-protein subunits. To date, 21 different mammalian G_α_ subunits, 6 G_β_ subunits and 12 G_γ_ subunits, several of which exist in alternatively spliced variants, have been identified (Simon et al., 1991; Neer, 1995; Oldham and Hamm, 2008). Gα subunits define the specificity of GPCR signal transduction and have been classified according to the degree of sequence homology into four families: G_α*s*_, G_α*i/o*_, G_α*q/11*_, and G_α12/13_ (Wettschureck and Offermanns, 2005). The pertussis toxin-sensitive G_α*i/o*_ family comprises G_α*i*_ and G_αo_, of which G_αo_ is the most abundant in brain tissue. *In situ* hybridization and immunohistochemical studies have shown that high densities of G_αo_ protein are present in the frontal cortex, cerebellum, hypothalamus, hippocampus, and substantia nigra (Worley et al., 1986; Schüller et al., 2001). Accordingly, G_αo_ knockout mice have neurological defects such as seizures, hyperactivity, poor motor coordination and abnormal sexual behavior (Jiang et al., 1998; Choi et al., 2016).

Despite the involvement of G_αo_ in large part of the modulatory signaling in the brain (Wettschureck and Offermanns, 2005) and its large potential as therapeutic targets (Li et al., 2020), our understanding of G_αo_ distribution in different neuron populations and its organization in different neuronal compartments is limited. To this end, we employed quantitative pre- and post-embedding immunoelectron microscopic techniques to unravel subcellular localization patterns of G_αo_ in different cerebellar cell types. Interestingly, we found that this G protein subunit exhibited distinct subcellular localization patterns throughout the cerebellar cortex, suggesting its involvement in regulating specific signaling pathways in post- and pre-synaptic compartments.

## Materials and Methods

### Animals

Four adult male C57BL/6J mice obtained from Charles River Laboratories (Barcelona, Spain) and housed in the Animal House Facilities of the Universidad de Castilla-La Mancha (Albacete, Spain), were used in the present study. All mice were maintained in cages on a 12-h light/12-h dark cycle at 24°C and received food and water *ad libitum*. Care and handling of animals prior to and during experimental procedures was in accordance with European Union regulations (86/609/EC), and all protocols and methodologies were approved and supervised by the local Animal Care and Use Committee.

For histoblotting, mice were deeply anesthetized by intraperitoneal injection of ketamine/xylazine 1:1 (ketamine, 100 mg/Kg; xylazine, 10 mg/Kg), the cerebellum was dissected, frozen rapidly in liquid nitrogen and stored at −80°C. For immunohistochemistry at the light microscopic and electron microscopic level, mice were firstly deeply anaesthetized by intraperitoneal injection of ketamine-xylazine 1:1 (0.1 mL/kg). Once reflex activity was completely abolished, the heart was surgically exposed for perfusion fixation through the ascending aorta, first with 0.9% saline and then followed by ice-cold fixative containing 4% (w/v) paraformaldehyde with 0.05% (v/v) glutaraldehyde and ∼0.2% picric acid made up in 0.1 M phosphate buffer (PB, pH 7.4) for 15 min. After perfusion, brains were removed and immersed in the same fixative for 2 h at 4°C. Tissue blocks were washed thoroughly in 0.1 M PB. Coronal 60 μm thick sections were cut on a Vibratome (Leica V1000).

### Antibodies and Chemicals

The following primary antibodies were used: rabbit anti-G_α*o*_ polyclonal (ref#ab154001; Recombinant fragment corresponding to Human GNAO1 aa 104–338; Abcam, Cambridge, United Kingdom); monoclonal anti-GABA_*B1*_ (N93A/49; aa. 873–977 of rat GABA_*B1*_, cytoplasmic C-terminus, Q9Z0U4; NeuroMab, UC Davis/NIH, United States); and guinea pig anti-PV (GP-Af1000; AB_2571615; Frontier Institute Co., Japan). The specificity of the G_αo_ antibodies used for immunohistochemistry at the light and electron microscopic levels is outlined in detail in the controls section and Supplementary Figures 1, 2. The characteristics and specificity of the monoclonal antibody targeting GABA_*B1*_ has been described by the manufacturer^1^.

The secondary antibodies used were as follows: alkaline phosphatase (AP)-goat anti-rabbit IgG (H + L) and goat anti-mouse IgG (H + L) (1:5,000; Sigma-Aldrich, Sant Louis, MO, United States), biotinylated goat anti-mouse IgG and biotinylated goat anti-rabbit pig IgG (Vector Laboratories, Burlingame, CA), anti-rabbit Alexa Fluor^®^-488 and anti-guinea pig Alexa Fluor^®^-594 (1:500; Jackson ImmunoResearch, Cambridge, United Kingdom), as well as goat anti-rabbit and anti-mouse IgG coupled to 1.4 nm gold (1:100; Nanoprobes Inc., Stony Brook, NY, United States).

### Histoblotting

The expression pattern and regional distribution of G_αo_ was analyzed in mice brains, using the histoblot technique (Aguado and Luján, 2019). Briefly, horizontal cryostat sections (10 μm) were overlapped with nitrocellulose membranes moistened with 48 mM Tris-base, 39 mM glycine, 2% (w /v) sodium dodecyl sulfate and 20% (v /v) methanol for 15 min at room temperature (∼20°C). After blocking in 5% (w /v) non-fat dry milk in phosphate-buffered saline for 1 h, nitrocellulose membranes were treated with DNase I (5 U /mL), washed and incubated in 2% (w /v) sodium dodecyl sulfate and 100 mM β-mercaptoethanol in 100 mM Tris–HCl (pH 7.0) for 60 min at 45°C to remove adhering tissue residues. After extensive washing, the blots were reacted with affinity-purified anti-G_αo_ antibodies (0.5 mg /mL) in blocking solution overnight at 4°C. The bound primary antibodies were detected with alkaline phosphatase-conjugated anti-rabbit IgG secondary antibodies for 2 h (Aguado and Luján, 2019). All nitrocellulose membranes were processed in parallel **and under the same conditions**. Digital images were acquired by scanning the nitrocellulose membranes using a desktop scanner (HP Scanjet 8300). Image analysis and processing were performed using the Adobe Photoshop software (Adobe Systems, San Jose, CA, United States) as described previously (Martín-Belmonte et al., 2020).

### Immunohistochemistry for Light Microscopy

Immunohistochemical reactions at the light microscopic level were carried out using the immunoperoxidase method as described earlier (Luján et al., 1996). Briefly, sections were incubated in 10% normal goat serum (NGS) diluted in 50 mMTris buffer (pH 7.4) containing 0.9% NaCl (TBS), with 0.2% Triton X-100, for 1 h. Sections were incubated in anti-G_αo_ (1–2 μg/ml diluted in TBS containing 1% NGS) overnight at 4°C, followed by incubation in biotinylated goat anti-rabbit IgG (Vector Laboratories, Burlingame, CA, United States) diluted 1:200 in TBS containing 1% NGS for 2 h at room temperature. Sections were then transferred into avidin–biotin–peroxidase complex (ABC kit, Vector Laboratories, Burlingame, CA, United States). Bound peroxidase enzyme activity was revealed using 3, 30-diaminobenzidine tetrahydrochloride (DAB; 0.05% in TB, pH 7.4) as the chromogen and 0.01% H_2_O_2_ as the substrate. Finally, sections were air-dried and mounted prior to observation in a Leica photomicroscope (DM 2500) equipped with a digital imaging camera (Leica DFC 500).

### Immunohistochemistry for Confocal Microscopy

Sections were firstly blocked in 10% NGS made up in TBS for 1 h before incubation in a mixture of primary antibodies for G_αo_ and Parvalbumin (PV) in TBS containing 2% NGS at 4°C overnight. After several washes in TBS, the sections were incubated in a mixture of secondary antibodies (anti-rabbit Alexa Fluor^®^-488 for G_αo_ and anti-rabbit Alexa Fluor^®^-594 for PV) made up in TBS, for 2 h at room temperature. After further washes in TBS, sections were mounted and coverslipped with fluorescence mounting medium (Vectashield, Vector Laboratory). Immunofluorescence labeling was examined using a confocal laser-scanning microscopy (Zeiss LSM 510-Meta, Jena, Germany). Separate color channels were acquired sequentially to avoid crosstalk between fluorochromes.

### Immunohistochemistry for Electron Microscopy

Immunohistochemical reactions at the electron microscopic level were carried out using the pre-embedding and post-embedding immunogold methods as described earlier (Luján et al., 1996). Ultrastructural analyses were performed in a Jeol-1010 (Tokyo, Japan) transmission electron microscope and image acquisition was done using a digital Gatan camera ES100W model 785 (Gatan, Inc., Pleasanton, CA, United States).

#### Pre-embedding Immunogold Method

Free-floating sections were first incubated in 10% NGS diluted in TBS, then incubated in anti-G_αo_ antibodies (1–2 μg/ml diluted in TBS containing 1% NGS) at 4°C overnight, followed by incubation in goat anti-rabbit IgG coupled to 1.4 nm gold (Nanoprobes Inc., Stony Brook, NY, United States), for 2 h at room temperature. Next, sections were postfixed in 1% glutaraldehyde and washed in double distilled water, followed by silver enhancement of the gold particles with a HQ Silver kit (Nanoprobes Inc.). Sections were then treated with osmium tetroxide (1% in 0.1 M PB), block-stained with uranyl acetate, dehydrated in graded series of ethanol and flat-embedded on glass slides in Durcupan (Fluka) resin. Regions of interest were cut at 70–90 nm on an ultramicrotome (Reichert Ultracut E, Leica, Vienna, Austria) and collected on single slot copper grids. Staining was performed on drops of 1% aqueous uranyl acetate followed by Reynolds’s lead citrate.

#### Post-embedding Immunogold Method

Ultrathin sections 80 nm thick from Lowicryl-embedded blocks of cerebellum were picked up on single slot coated nickel grids and incubated on drops of a blocking solution consisting of 2% Human Serum Albumin (HSA) in 0.05 M TBS and 0.03% (v / v) Triton X-100 (TBST) at 4°C overnight. For single labeling, the grids were incubated with anti-G_αo_ antibodies (10 μg/ml in TBST with 2% HSA) at 28°C overnight and then on drops of goat anti-rabbit IgG conjugated to 10 nm colloidal gold particles (Nanoprobes) in 2% (w / v) HSA and 0.5% (w / v) polyethylene glycol in TBST, for 2 h at room temperature. For double labeling, the grids were incubated with anti-G_α*o*_ antibodies (10 μg/ml in TBST with 2% HSA) and anti-GABA_*B1*_ (10 μg/ml in TBST with 2% HSA) at 28°C overnight and then on drops of goat anti-rabbit IgG conjugated to 10 nm colloidal gold particles and goat anti-mouse IgG conjugated to 20 nm colloidal gold particles (Nanoprobes) in 2% (w / v) HSA and 0.5% (w / v) polyethylene glycol in TBST, for 2 h at room temperature. Finally, the grids were washed in TBS and counterstained for electron microscopy with saturated aqueous uranyl acetate followed by lead citrate.

### Quantification of G_αo_ Immunoreactivity in Cerebellar Neurons

To establish the relative abundance of G_αo_ immunoreactivity in different compartments of cerebellar neurons, we used 60 μm-thick coronal slices processed for pre-embedding immunogold immunohistochemistry and 100 nm-thick ultrathin sections processed for post-embedding immunogold immunohistochemistry, as described previously (Luján et al., 1996). Briefly, three samples of tissue were obtained for each of three mice. Electron microscopic serial ultrathin sections were cut close to the surface of each block to minimize false negatives due to reduction of immunolabeling with depth. Quality of immunolabeling was evaluated by selecting areas with immunogold particles at approximately the same distance from the tissue surface. Randomly selected areas were then photographed from the selected ultrathin sections at a magnification of 35,000 X. Quantification of immunogold labeling was carried out in reference areas totaling approximately 3,000 μm^2^, and performed in three different ways:

#### Percentage of Immunoparticles for G_αo_

To study the frequency of G_αo_ in the molecular layer and the granule cell layer, the immunoparticles identified in each reference area and present in the different subcellular compartments; dendritic spines, dendritic shafts and axon terminals, was counted. The data were expressed as a percentage of immunoparticles at a given compartment, both in the plasma membrane and at intracellular sites. In addition, using the post-embedding immunogold, the numbers of gold particles associated with postsynaptic densities (PSDs), and in addition along the extrasynaptic plasma membrane and intracellular sites in dendritic spines of PCs, were also counted. Given the low frequency of finding spines attached through their neck to parent shafts in ultrathin sections, immunoparticles were not counted on the necks of spines. Finally, the percentage of gold particles in the three subcompartments was calculated. Background labeling was calculated in the molecular layer and granule cell layer by counting the immunoparticles for G_αo_ detected in mitochondria, myelinated axons, or lumen of capillary, as examples of location where G_αo_ is not expected to be. From a total of 7,308 immunoparticles counted, only 22 (0.3%) were detected in non-specific sites. Given this very low number of background labeling, we did not subtract it in our analyses.

#### Distribution of G_αo_ Relative to Glutamate Release Sites

To determine the relative abundance of G_αo_ in dendritic spines of PCs, and their association with excitatory synapses, immunoparticles identified in each reference area and present in dendritic spines were counted. Using pre-embedding and post-embedding immunogold techniques, excitatory synapses were identified by the presence of: (i) a PSD, (ii) synaptic vesicles at the presynaptic terminal, and (iii) opposing membranes between the pre and the post-synaptic terminals. As differences in the distribution of immunoparticles among all samples were not statistically significant (*P* > 0.56, *Kolmogorov–Smirnov non-parametric test*) the data were pooled. The length of the dendritic spine membrane from the edge of the synaptic junction and the length from the center of each immunoparticle present was then measured along the plasma membrane to the edge of the postsynaptic density, using a digitizing tablet and appropriate software (ImageJ). Finally, the number of immunoparticles was expressed as relative frequency in 60 nm membrane segments of spine membrane, thus obtaining a normalized value of the relative abundance of G_αo_ along the PC spines.

To establish the relative abundance of G_αo_ relative to glutamate release sites in granule cell dendrites located in the glomeruli, all dendritic shafts establishing synapses with mossy fibers were counted and assessed for the presence of immunoparticles. The distribution of gold particles among different samples were not statistically significant (*P* = 0.74, Kolmogorov-Smirnov non-parametric test), and thus all data was pooled. The extrasynaptic membrane from all immunopositive dendrites was measured using a digitizing tablet and appropriate software (Sigma-Scan Pro, Jandel Scientific, Erkrath, Germany), and then divided into 60 nm bins, within the first 300 nm of the plasma membrane from a mossy fiber synapse. Finally, the number of immunoparticles was expressed as relative frequency in bins corresponding to 60 nm membrane segments of dendrite membrane (only within the first 300 nm of plasma membrane) and gold particles present beyond 300 nm were pooled to obtain a normalized value of the relative abundance of G_αo_ along granule cell dendrites.

#### Tangential Distribution of G_αo_ Along PSDs

To establish the tangential distribution of G_αo_ at PSDs of excitatory synapses in spines of PCs, quantification of immunolabeling was performed from ultrathin sections processed for post-embedding immunogold, as described earlier (Lin et al., 2008). Briefly, ultrathin sections picked up on pioloform-coated mesh grids were immunoreacted for G_αo_ and photographed from three animals. Postsynaptic specializations were included in the analysis only if the synaptic cleft was visible, omitting synapses cut tangentially. The distance between the midline and the edge of the PSD was divided into five bins, each bin corresponding to 10% of the PSD length in a single section, and each gold particle was assigned to a bin. The distribution obtained was mirrored across the midline for display. The data was expressed as percentage of immunoparticles along the PSD length.

### Controls

For analyzing antibody specificity, we used the approach outline elsewhere (Rhodes and Trimmer, 2008), as no G_αo_ knockout mice were available. Labeling patterns are compared using different antibodies raised against non-overlapping epitopes on the same target protein (Supplementary Figures 1, 2). We found similar patterns at both the light (Supplementary Figure 1) and electron microscopic level (Supplementary Figure 2), indicating that the signal was representative of specificity of the antibody. In addition, we compared the mRNA expression pattern of Gnao1 gene using the Allen Brain Atlas^2^ with the immunohistochemical pattern of G_αo_ obtained with our antibodies. *In situ* hybridization showed strong signal for the Gnao1 gene in the cortex, followed by the hippocampus, striatum and cerebellum, with lower expression pattern in the thalamus. In the cerebellum, transcript for Gnao1 were detected at high levels in the soma of Purkinje cells, with lower levels in interneurons distributed through the molecular layer, and granule cells and Golgi cells in the granule cell layer. Transcripts for Gnao1 can also be seen in scattered cells among Purkinje cells, likely representing Bergmann glia. This mRNA expression pattern is fully consistent with the immunohistochemical pattern described in this manuscript. Furthermore, the primary antibody was either omitted or replaced with 5% (v/v) normal serum of the species of the primary antibody, resulting in total loss of the signal. For the pre-embedding technique, labeling patterns were also compared with those obtained by Calbindin (polyclonal rabbit anti-calbindin D-9k (CB9; Swant, Marly, Switzerland); only the antibodies against G_αo_ consistently labeled plasma membranes.

## Results

### Expression and Distribution of G_αo_ in the Cerebellum

**FIGURE 1:**
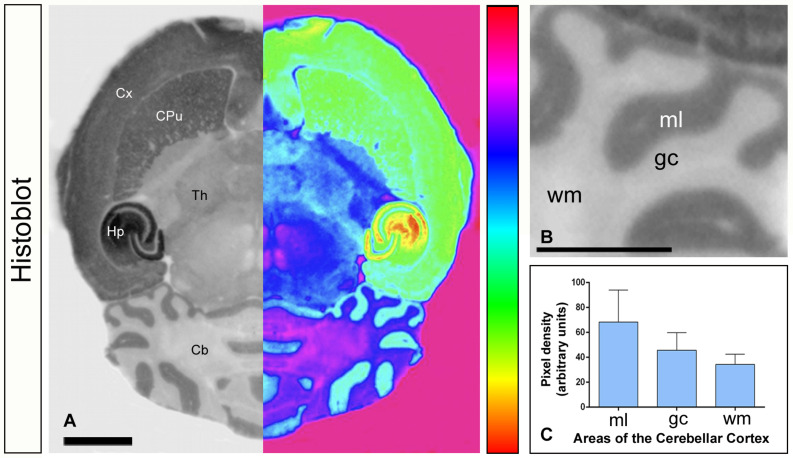
Expression of G_αo_ in the brain. **(A–C)** The protein expression of G_αo_ was visualized in histoblots of horizontal brain sections in adult mice using an affinity-purified anti-G_αo_ antibody. The expression of G_αo_ revealed marked region-specific differences, with strongest immunoreactivity in the hippocampus (Hp), followed by cortex (Cx), caudate putamen (CPu) and cerebellum (Cb). Moderate expression level was detected in the thalamus (Th) [panel **(A)**]. For illustration purposes, greyscale histoblot images were converted to color gradients using gradient mapping [panel **(A)**]. In the cerebellum, densitometric analysis showed strong immunoreactivity for G_αo_ in the molecular layer (ml), with lower intensity in the granule cell layer (gc) and very weak in the white matter (wm) [panels **(B,C)**]. Error bars indicate SEM. Scale bars: **(A)** 2 mm; **(B)** 1 mm.

**FIGURE 2:**
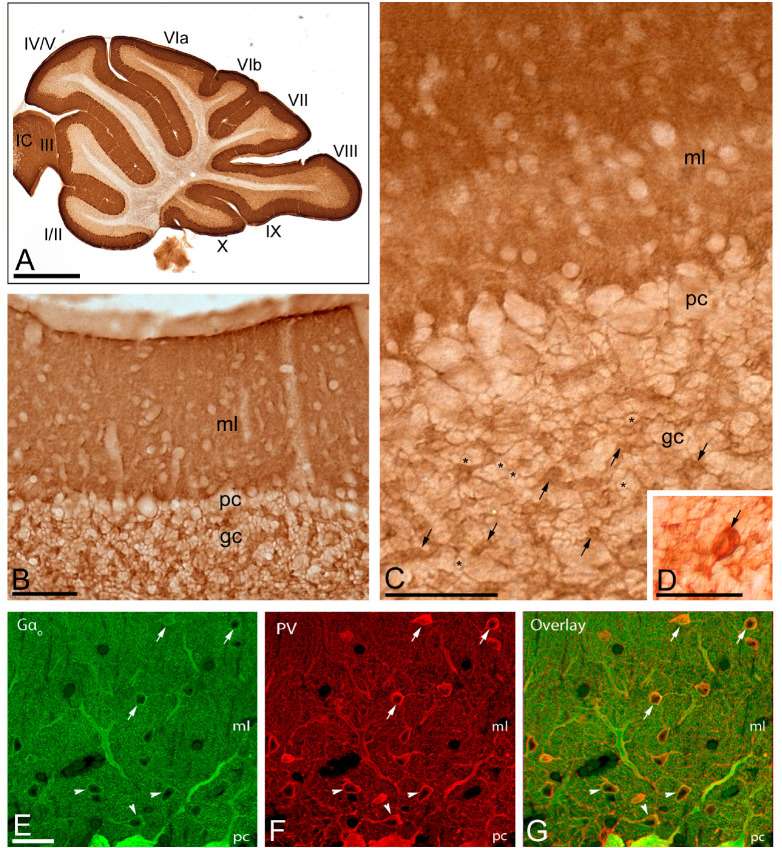
Regional and cellular distribution of G_αo_ in the cerebellum. **(A–D)** Immunoreactivity for G_αo_ in the cerebellar cortex using an immunoperoxidase method. The parasagittal photomicrography of the cerebellum [panel **(A)**] shows strong immunolabeling for G_αo_ in the molecular layer (ml), in somata of PCs in the Purkinje cell layer (pc) and moderate in the granule cell layer (gc), with similar distribution pattern and expression levels in all cerebellar lobules (lobules I to X). Immunoreactivity for G_αo_ was detected in the neuropil of the molecular layer, but outlined granule cells (example, asterisks), and labeled glomeruli (example, arrows) and Golgi cells [arrow in panel **(D)**]. IC: inferior colliculus. **(E–G)** Co-localization of G_αo_ with Parvalbumin (PV) in basket cells (arrowheads) and stellate cells (arrows) using double-labeling immunofluorescence visualized with scanning confocal microscopy. Confocal images of the cerebellar cortex showing immunofluorescent labeling for G_αo_ (left, green) and PV (middle, red) and their colocalization in the overlay image (right). *Abbreviations*: ml, molecular layer; pc, Purkinje cell layer; gc, granule cell layer. Scale bars: **(A)** 1 mm; **(B–D)** 10 μm; **(E–G)** 20 μm.

We first investigated the expression levels of G_αo_ in the brain using a G_αo_ subunit-specific antibody in conventional histoblots (Figures 1A–C). The brain expression of G_αo_ revealed region-specific differences, with strongest immunoreactivities in the hippocampus, caudate putamen, septum, cortex and cerebellum, and moderate labeling in the thalamus and midbrain nuclei (Figure 1A). In the cerebellum, immunostaining for G_αo_ was significantly stronger in the molecular layer and Purkinje cell layer than in the granule cell layer, in which moderate to weak labeling was found (Figures 1B,C). The white matter consistently showed very weak G_αo_ staining (Figures 1B,C). Light microscopic examination by immunoperoxidase methods revealed a similar distribution pattern along all cerebellar lobules, from rostral to caudal (Figure 2A), with a strong localization on the neuropil of the molecular layer (Figures 2A–C). At the cellular level, labeling for G_αo_ could be seen outlining the somata of PCs (Figure 2C), as well as outlining the somata of basket and stellate cells in the molecular layer (Figures 2E–G). In the granule cell layer, G_αo_ immunolabeling was present in GCs and glomeruli (Figures 2B,C), and in Golgi cells (Figure 2D). Taken together our light microscopic examination revealed that G_αo_ is widely expressed in the adult brain and in several neuron populations in the cerebellar cortex.

### Subcellular Localization of G_αo_ in Purkinje Cells

**FIGURE 3:**
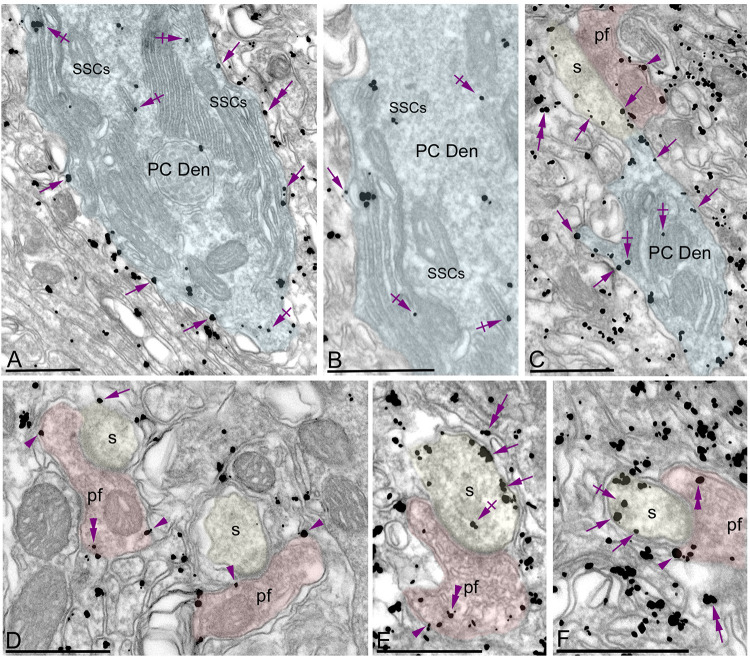
Subcellular localization of G_αo_ in PCs. Electron micrographs showing immunoparticles for G_αo_ in the molecular layer of the cerebellum, as detected using a pre-embedding immunogold technique. **(A–F)** Most immunoparticles for G_αo_ were distributed along the extrasynaptic plasma membrane (arrows) of dendritic shafts (Den, blue transparent overlay) and spines (s, yellow transparent overlay) of PCs contacted by parallel fiber terminals (pf, red transparent overlay). To a lesser extent, immunoparticles for G_αo_ were detected at intracellular sites (crossed arrows) associated with intracellular membranes and more frequently with subsurface cisterns (SSCs) belonging to the endoplasmic reticulum. Presynaptically, G_αo_ immunoparticles were also distributed along the plasma membrane and at intracellular sites (arrowheads) in axon terminals of parallel fibers (pf). In addition to PCs, immunoparticles for G_αo_ were detected in Bergmann glia cells (double arrows). Scale bars: **(A–F)** 500 nm.

To investigate the subcellular localization of G_αo_ in cerebellar neurons, we performed electron microscopic studies using the pre-embedding immunogold technique combined with quantitative analyses in the cerebellar cortex. In the molecular layer, immunoreactivity for G_αo_ was mainly detected postsynaptically in dendritic shafts and spines of PCs associated with the plasma membrane (Figures 3A–F). Less frequently, immunoparticles for G_αo_ were found at intracellular sites (Figures 3A–F). Most of these immunoparticles were associated with cisterns running in parallel to the inner leaflet of the plasma membrane, and identified as subsurface cisterns (SSCs), which belong to the endoplasmic reticulum (ER) and are free of ribosomes (Figures 3A,B). Presynaptically, a low number of G_αo_ immunoparticles were observed in parallel fiber terminals establishing excitatory synapses with spines of PCs (Figures 3C–F). In addition to PCs, immunoreactivity for G_αo_ was also observed in profiles lacking many organelles and surrounding PC-parallel fiber synapses, which were identified as Bergman glia cells (Figures 3A,C,E,F).

**FIGURE 4:**
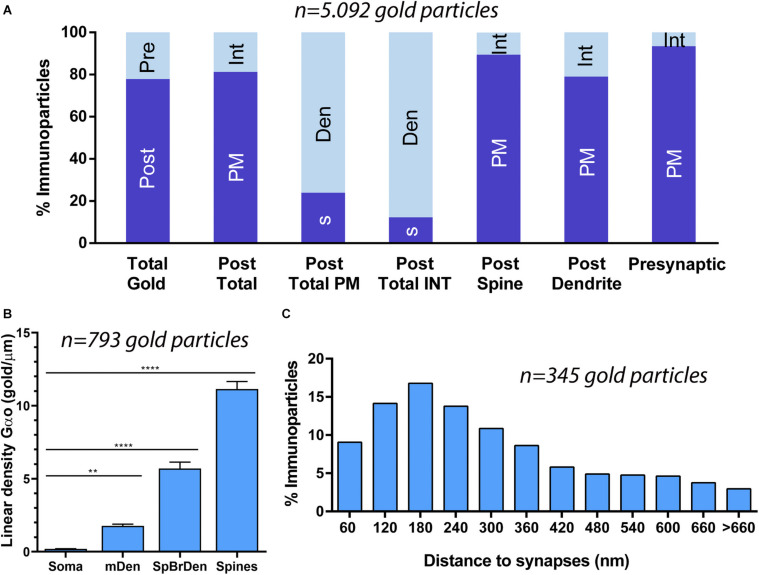
Compartmentalization of G_αo_ in PCs. **(A)** Histogram showing the percentage of immunoparticles for G_αo_ in neuronal compartments in the molecular layer. A total of 5,092 immunoparticles in the molecular layer were analyzed, of which 78% were postsynaptic and 22% were presynaptic. Immunoparticles for G_αo_ at postsynaptic sites were found in PC dendrites (76.08%) and in PC spines (23.92%), being mainly distributed along the plasma membrane (79.02% in dendrites and 89.44% in spines) and intracellularly (20.98% in dendrites and 10.56% in spines). Immunoparticles for G_αo_ at presynaptic sites were detected in parallel fiber terminals mostly distributed along the plasma membrane (93.43%) and to a lesser extent at cytoplasmatic sites (6.57%). **(B)** Somato-dendritic gradient of G_αo_ along the surface of PCs showing that density of immunoparticles increased significantly from soma to dendritic spines. Error bars indicate SEM; ** *P* < 0.002; **** *P* < 0.0001. mDen, main dendrites; SpBrDen, spiny branchlet dendrites. **(C)** Histogram showing the proportion of immunoparticles for G_αo_ at a given distance from the edge of PSDs in PC spines-parallel fiber synapses. The data shows that 65% of G_αo_ were distributed in a 60–300 nm wide band, indicating its proximity to excitatory synapses.

Quantitative analysis performed on the neuropil showed that from 5,092 immunogold particles analyzed, 3,965 (77.87%) were distributed at postsynaptic compartments and 1,127 (22.13%) at presynaptic sites (Figure 4A). Postsynaptically, 23.92% of immunoparticles for G_αo_ were found in PC spines and, of those, 89.44% were distributed along the plasma membrane and 10.56% at intracellular sites (Figure 4A). In dendritic shafts of PCs, which contained 76.08% of all G_αo_ immunoparticles, 79.02% were distributed along the plasma membrane and 20.98% at intracellular sites (Figure 4A). Presynaptically, 93.43% of immunoparticles for G_αo_ were distributed along the plasma membrane and 6.57% at intracellular sites (Figure 4A).

To determine the abundance of G_αo_ along the surface of PCs, we analyzed the plasma membrane distribution as a function of distance from the soma (Figure 4B). Quantitative analysis of immunogold distribution in four somato-dendritic domains of PCs showed a distance-dependent increase of G_αo_ from soma to dendritic spines (Figure 4B). Thus, density was low in the somata (0.20 ± 0.01 immunoparticles/μm^2^), increased in the main dendrites in the molecular layer (1.78 ± 0.11 immunoparticles/μm^2^), and even more in the spiny branchlet dendrites (5.70 ± 0.44 immunoparticles/μm^2^) and was highest in dendritic spines (11.15 ± 0.51 immunoparticles/μm^2^) (Figure 4B). This approach demonstrated that G_αo_ followed a non-uniform distribution along the somato-dendritic surface of PCs.

Because of the high density of G_αo_ in PC spines, we assessed how this G protein subunit is distributed relative to the glutamate release site (Figure 4C). The distribution of G_αo_ immunoparticles (*n* = 345), normalized to relative frequency in 60 nm bins, showed a peak between 0 and 300 nm from the synapses, for 65% of all immunoparticles observed, with G_αo_ density decreased markedly further within the PC spine membrane (Figure 4C). This approach revealed that on dendritic spines of PCs, G_αo_ was preferentially localized around parallel fiber-PC excitatory synapses.

### Synaptic Localization of G_αo_ in Excitatory Synapses

**FIGURE 5:**
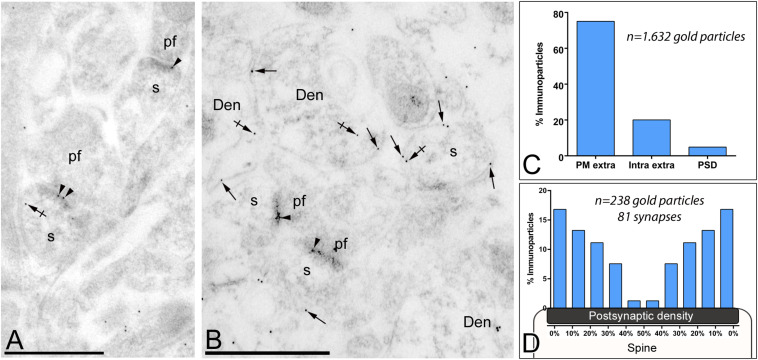
Subsynaptic localization of G_αo_ in PCs. Electron micrographs of the molecular layer of the cerebellum showing G_αo_ immunoparticles in excitatory synapses of PCs using a post-embedding immunogold method. **(A,B)** Immunoparticles for G_αo_ were observed in the PSD of PC spines (s) (arrowheads) establishing excitatory synapses with parallel fiber terminals (pf). They were also observed along the extrasynaptic plasma membrane (arrows) of PC spines (s) and PC dendrites (Den), and at intracellular sites (crossed arrows). **(C)** Histogram showing the percentage of immunoparticles for G_αo_ in different compartments of PC spines. A total of 1,632 G_αo_ immunoparticles were analyzed in PC spines, of which 5% were located in PSDs, 75% along the extrasynaptic plasma membrane and 20% at cytoplasmic sites. **(D)** Quantitative analysis showing the tangential distribution of immunoparticles for G_αo_ across the PSD. G_αo_ immunoparticles were preferentially located at the edges of the PSD, with the density decreasing toward the middle of the synapse. Scale bars: **(A,B)** 500 nm.

Previous immunoelectron microscopy studies have demonstrated that GABA_*B*_ receptors and GIRK channels are localized along the PSD of spines at parallel fiber-PC synapses (Kulik et al., 2002; Fernández-Alacid et al., 2009). To verify the possible location of G_αo_ within the PSD, we carried out post-embedding immunogold electron microscopy (Figure 5). We found G_αo_ immunoparticles in the main body of the postsynaptic density (PSD) of PC spines establishing synapses with parallel fiber terminals (Figure 5A), in addition along the extrasynaptic plasma membrane and cytoplasmic sites in dendritic spines and shafts (Figures 5A,B). Quantitative analysis demonstrated that synaptic localization of G_αo_ represented a small fraction (5% of all immunoparticles) compared to the main extrasynaptic localization (plasma membrane: 75% of all immunoparticles; intracellular: 20% of all immunoparticles) in spines and dendrites (Figure 5C). Within its synaptic localization, immunoparticles for G_αo_ decreased markedly toward the center of the junction and concentrate in the edges of the PSD (Figure 5D), suggesting a subsynaptic preference of the G-protein subunit.

### G_αo_ Co-localizes With GABA_*B*_ Receptors in Purkinje Cells

**FIGURE 6:**
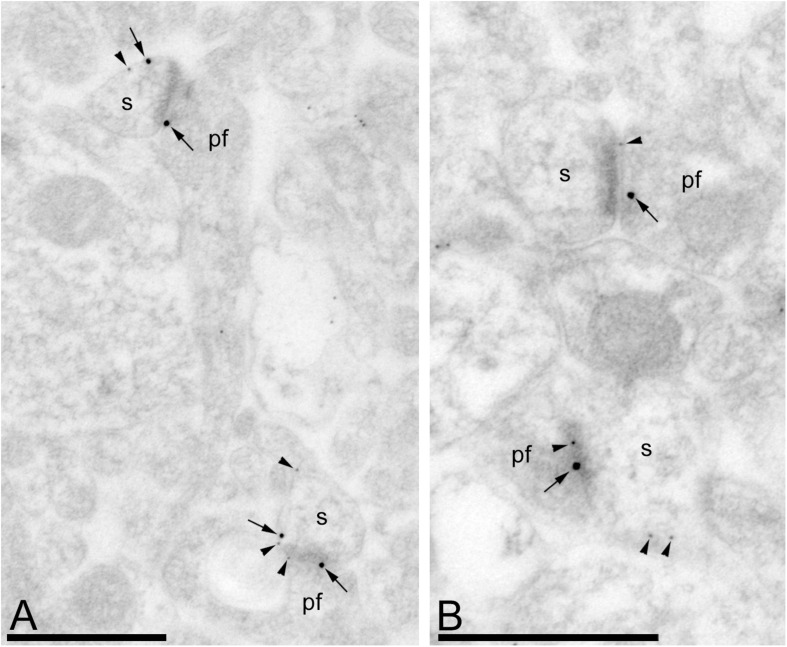
G_αo_ and GABA_*B1*_ reside in the same dendritic spine of PCs. Electron micrographs of the molecular layer of the mouse cerebellar cortex showing immunoreactivity for G_αo_ (10 nm particles, arrowheads) and GABA_*B1*_ (20 nm particles, arrows), as detected using a double-labeling post-embedding immunogold method. G_αo_ and GABA_*B1*_ were observed at PSDs of excitatory synapses and extrasynaptic sites between PC spines (s) and parallel fibers (pf), many times very close to each other. Scale bars: **(A,B)** 500 nm.

Previous studies have shown that GPCRs and effector ion channels are expressed in different neuronal populations of the cerebellum (Aguado et al., 2008; Ciruela et al., 2010). To determine whether G_αo_ are present in the same compartment of PCs expressing GABA_*B*_ receptors, double label post-embedding immunogold electron microscopy was performed (Figure 6). Throughout the molecular layer, immunoparticles for G_αo_ were detected in the PSD as well as in the extrasynaptic plasma membrane that also labeled for GABA_*B1*_ (Figures 6A,B). Due to the spatial resolution of the post-embedding technique, of around 26 nm when using 20-nm immunoparticles or around 21 nm when using 10-nm immunoparticles, a distance wider than the synaptic cleft, some of the immunoparticles for GABA_*B1*_ were detected on the synaptic cleft (Figure 6A) or immediately on the plasma membrane of the axon terminal (Figure 6B). Regardless of this technical limitation, immunoparticles for G_αo_ and GABA_*B1*_ were detected in close proximity both at extrasynaptic and synaptic sites (Figures 6A,B). Therefore, G_αo_ and GABA_*B1*_ cohabit the same microdomain within different subcompartments of dendritic spines.

### Subcellular Localization of G_αo_ in Stellate and Basket Cells

**FIGURE 7:**
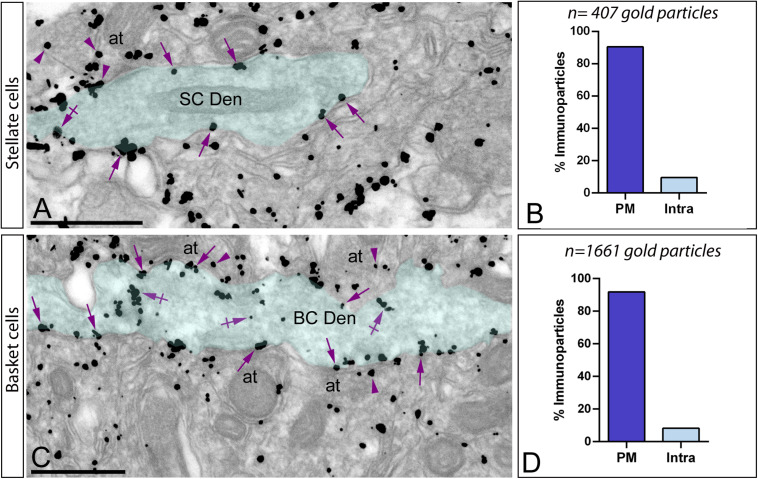
Subcellular localization of G_αo_ in basket and stellate cells. Electron micrographs showing immunoparticles for G_αo_ in the molecular layer of the cerebellum, as detected using a pre-embedding immunogold technique. **(A,B)** In the outer two-thirds of the molecular layer, immunoparticles for G_αo_ were distributed in smooth dendritic shafts receiving excitatory synapses on their surface, identified as stellate cells (SC Den, green transparent overlay). From a total of 407 G_αo_ immunoparticles analyzed, 90.4% were located along the plasma membrane (PM) (arrows) and only 9.6% at intracellular sites (Intra) (crossed arrows). **(C,D)** In the inner third of the molecular layer, immunoparticles for G_αo_ were distributed in smooth dendritic shafts receiving excitatory synapses on their surface, identified as basket cells (BC Den, green transparent overlay). From a total of 1.661 G_αo_ immunoparticles analyzed in basket cell dendrites, 91.81% were located along the PM (arrows) and only 8.19% at intracellular sites (Intra) (crossed arrows). Immunoparticles for G_αo_ were also found in axon terminals (arrowheads) (at) establishing excitatory synapses with basket cell dendrites. Scale bars: **(A,C)** 500 nm.

In addition to PCs, immunoparticles for G_αo_ were localized in stellate cells (Figures 7A,B) in basket cells (Figures 7C,D), which were identified based on their smooth dendritic shafts receiving multiple symmetrical and asymmetrical synapses on the surface and their location in the outer two thirds and the inner third of the molecular layer, respectively. Immunoparticles for G_αo_ were also found in axon terminals establishing excitatory synapses with basket cell dendrites (Figures 7A,C). Quantitative analysis performed on the dendrites of stellate cells revealed that from 407 immunoparticles analyzed, 368 (90.4%) were distributed along the plasma membrane and 39 (9.6%) were found at cytoplasmic sites associated with intracellular membranes (Figure 7B). Similarly, from 1,661 immunogold particles analyzed, 1,525 (92%) were distributed along the plasma membrane and 136 (8%) were found at cytoplasmic sites associated with intracellular membranes (Figure 7D).

### Subcellular Localization of G_αo_ in the Granule Cell Layer

**FIGURE 8:**
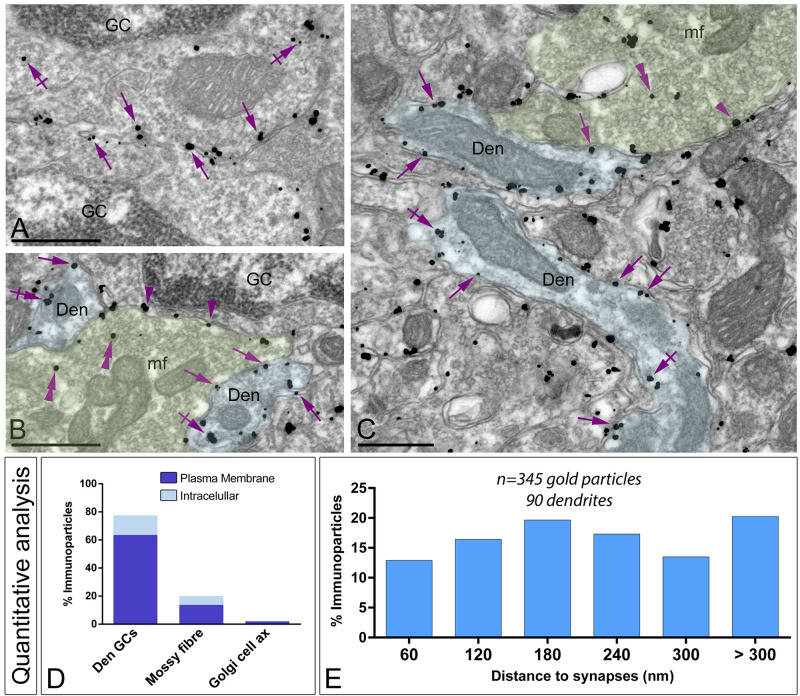
Subcellular localization of G_αo_ in the granule cell layer. Electron micrographs showing immunoparticles for G_αo_ in different neuronal elements present in the granule cell layer of the cerebellum, as detected using a pre-embedding immunogold technique. **(A–C)** Immunoparticles for G_αo_ were found along the somatic plasma membrane (arrows) and intracellular sites (crossed arrows) of granule cells (GC). Outside granule cell somata, G_αo_ immunoparticles were detected in glomeruli, mainly distributed in the plasma membrane (arrows) and intracellularly (crossed arrows) in granule cell dendrites (Den, blue transparent overlay), as well as presynaptically in mossy fibre terminals (mf, green transparent overlay) in the plasma membrane (arrowheads) and intracellularly (double arrowheads). Scale bars: **(A–C)**, 500 nm. **(D)** Histogram showing the percentage of immunoparticles for G_αo_ in glomeruli. A total of 2,194 immunoparticles were analyzed, of which 77.53% were detected in GC dendrites (Den GCs), 20.10% in mossy fiber terminals and only 2.37% in axons of Golgi cells, being mainly distributed along the plasma membrane. **(E)** Histogram showing the proportion of immunoparticles for G_αo_ (*n* = 346 immunoparticles on 90 dendrites) relative to the release of glutamate GC dendrites-mossy fiber synapses. The data shows that 80% of G_αo_ were distributed in a 60–300 nm wide band, indicating its proximity to mossy fiber synapses.

The subcellular localization of G_αo_ in the granule cell layer was determined on samples taken from areas with only GCs and areas with cerebellar glomeruli (Figures 8A–E). Immunoreactivity for G_αo_ was found in the somatic plasma membrane of GCs (Figures 8A,B), and in addition along the plasma membrane and intracellular sites of the three neuronal elements forming the cerebellar glomeruli (Figures 8B,C). To determine how G_αo_ is organized in glomeruli, quantitative analysis was performed. Data revealed that immunoparticles were primarily associated with dendrites of GCs (63.58% along the plasma membrane and 13.95% at intracellular sites; *n* = 1,701 particles in 270 dendrites), establishing excitatory synapses with mossy fibers (Figure 8D). Immunoparticles for G_αo_ were also present presynaptically in mossy fiber terminals (13.72% along the plasma membrane and 6.38% at intracellular sites; *n* = 441 particles in 37 terminals) (Figure 8D). Weak immunoreactivity for G_αo_ was detected in Golgi cell axon terminals (1.96% along the plasma membrane and 0.41% at intracellular sites; *n* = 43 particles in 28 terminals) establishing symmetrical synapses with dendrites of GCs (Figure 8D).

To gain further insights into the subcellular compartmentalization of G_αo_ in the dendrites of GCs, we analyzed its distribution in relation to the glutamate release site (Figure 8E). Analyzing the position of G_αo_ immunoparticles (*n* = 346 on 90 dendrites) in relation to the closest edge of the postsynaptic membrane specialization, we determined that 80% of the immunoparticles for G_αo_ were present close to the GC dendrite–mossy fiber synapse, within a distance of 300 nm from the edge of mossy fiber synapses (Figure 8E). This data indicates an enrichment of G_αo_ in the vicinity of excitatory synapses on GC dendrites.

## Discussion

Despite the importance of G proteins and upstream GPCRs in defining functions of cerebellar circuits, the cellular and subcellular localization of many G protein subunits remains mostly elusive. G_αo_ is one of the most abundant G-protein subunits in the brain, but its functions are poorly understood. The identification of the subcellular compartments and cell types where G-protein subunits are functional could be associated to physiological characteristics to better explain the structure-function relationship of G_αo_. Thus, taking advantage of pre- and post-embedding immunoelectron microscopy we demonstrated a major postsynaptic and occasionally presynaptic localization of G_αo_ in the cerebellar cortex. Quantitative analyses revealed a large proportion of G_αo_ on extrasynaptic plasma membrane in several cerebellar neurons, in addition to be expressed along PSDs of glutamatergic synapses between PC spines and parallel fiber terminals. Here we show the first detailed description of the subcellular localization of G_αo_ in cerebellar circuits, providing insights into cell- and neuronal compartment-specific roles of G_α*o*_.

### Differential Expression and Cell Type-Specific Localization of G_αo_ in the Cerebellar Cortex

Determining the brain expression pattern of G_αo_ is an important step to unraveling the molecular basis of its signaling in different regions. Using the histoblot technique, we demonstrated region-specific differences in the protein expression patterns of G_αo_ in the brain, consistent with earlier *in situ* hybridization studies and autoradiographic studies (Worley et al., 1986; Schüller et al., 2001; Cha et al., 2019). Although widely expressed, important differences in G_αo_ were observed between different brain regions. Such disparity may reflect G protein expression within individual cell types, as well as innervation patterns to specific brain regions.

Mice lacking G_αo_ highlighted the crucial role of this G protein subunit in the correct development of the cerebellar cortex, which affected mainly the PCs and GCs (Cha et al., 2019). Consistent with this observation, we found that G_αo_ is distributed in many cerebellar neurons, i.e., PCs, GCs, basket cells, stellate cells, and Golgi cells. Previous studies have examined the cerebellar distribution of G_αo_ subunit protein (Schüller et al., 2001; Cha et al., 2019). Although the data obtained in these reports are fully in agreement with those presented here, our data provide a more detailed and comprehensive cellular map of the G_αo_ distribution, extending the list of cell types expressing G_αo_ in the cerebellum.

### Preferential Localization of G_αo_ on Somato-Dendritic Compartments of Cerebellar Neurons

At the light microscopic level, our data indicated a widespread distribution of G_αo_ throughout the cerebellar cortex. The use of pre-embedding immunogold revealed that immunolabeling detected in the molecular layer of the cerebellar cortex is attributed to the abundance of G_αo_ in PCs, where most immunoparticles localized to the cytoplasmic face of the plasma membrane. At this location, they function as relay proteins between membrane-bound GPCRs, including GABA_*B*_ receptors, and their target signaling molecules (Wettschureck and Offermanns, 2005; de Oliveira et al., 2019). Our ultrastructural localization of immunoreactivity for G_αo_ is in line with immunohistochemical studies reporting the high levels of G_αo_ in the cerebellar cortex (Worley et al., 1986; Schüller et al., 2001), although we extend previous data providing a precise subcellular localization pattern of G_αo_ en different cerebellar neurons and cellular compartments.

In PCs, the majority of G_αo_ immunoparticles were localized to spines, postsynaptic to glutamatergic parallel fiber axon terminals, mainly at extrasynaptic sites using pre-embedding immunogold approaches, consistent with the reported subcellular localization of GABA_*B*_ receptors (Kulik et al., 2002; Fernández-Alacid et al., 2009). However, when the pre-embedding immunogold method is applied to molecules present at the PSD of excitatory synapses, their detection is hampered (Luján et al., 1996; Fritschy et al., 1998; Watanabe et al., 1998). These difficulties are overcome using the post-embedding immunogold method (Fritschy et al., 1998). Therefore, we applied this approach, resulting in labeling for G_αo_ along the main body of PC-parallel fiber synapses. Consistent with our data, proteomic studies using a TAP-tagged PSD-95 knockin mice showed that G_αo_ appears in postsynaptic densities *in vivo* (Fernández et al., 2009). Our quantitative analysis showed that this synaptic location of G_αo_ represented a small proportion compared to that located along the extrasynaptic plasma membrane and cytoplasmic sites. In agreement with previous studies using similar quantitative immunohistochemical approaches that reported the presence of GABA_*B*_ receptors in the same synaptic compartments (Kulik et al., 2002; Fernández-Alacid et al., 2009), we further revealed using double labeling immunogold that GABA_*B*_ receptors and G_αo_ occupied the same PSD in PC-parallel fiber synapses. Furthermore, the combination of two gold particles of different size allowed us to demonstrate that G_αo_ and GABA_*B*_ receptors are also spatially close to each other. Compiling evidence showed that GABA_*B*_ receptors form macromolecular complexes with G proteins and downstream effectors (Clancy et al., 2005; Fowler et al., 2007; Fernández-Alacid et al., 2009; Ciruela et al., 2010; Laviv et al., 2011; Fajardo-Serrano et al., 2013; Schwenk et al., 2016). Thus, it seems reasonable to think that the G_αo_ subunit acts to regulate the activity of GABA_*B*_ receptors at synaptic and extrasynaptic sites in PCs, in proximity forming macromolecular complexes, where other combining molecules like effector channels and regulatory proteins can be present in the signalosome.

In contrast to the large proportion of G_αo_ around glutamatergic synapses in PCs, immunolabeling for G_αo_ was random around dendritic shafts of stellate cells, basket cells and GCs, three neuron types known to express GABA_*B*_ receptors (Billinton et al., 1999; Bischoff et al., 1999; Ige et al., 2000). Our quantitative analysis demonstrated a uniform distribution on dendritic shafts of GCs, with no preference in the G_αo_ localization around excitatory synapses established by mossy fibers. In agreement with this distribution pattern, previous studies established that GABA_*B*_ receptors show a similar subcellular localization in GC dendrites (Ciruela et al., 2010). Altogether, the ubiquitous distribution of G_αo_ in many cerebellar cells suggests the involvement of this G-protein subunit in most cerebellar circuits. The close spatial and functional relation of G_αo_ and GABA_*B*_ receptors in the same compartments of those neurons remains to be demonstrated.

### Additional Subcellular Localizations of G_αo_ in the Cerebellar Cortex

In addition to the preferential localization of G_αo_ on somato-dendritic compartments of PCs, basket cells, stellate cells and GCs, our ultrastructural data also revealed its presence presynaptically in axon terminals from different sources including parallel fibers, mossy fibers, and Golgi cell axons. Existing evidence has shown that signaling through G_*i/o*_ mediate presynaptic inhibitory effects on neurotransmitter release in axon terminals (Brown and Sihra, 2008). Our immunogold labeling showing association of G_αo_ with the active zone and extrasynaptic membrane of axon terminals establishing asymmetrical synapses is virtually identical to that described for GABA_*B*_ receptors (Fernández-Alacid et al., 2009). These data suggest the involvement of G_αo_ in the regulation of neurotransmitter release in the cerebellum, although a direct demonstration is needed. Activation of presynaptic G_*i/o*_-coupled GABA_*B*_ receptors supress glutamate release in presynaptic PF terminals by dampening Ca^2+^ influx *via* P/Q-type and N-type voltage-gated Ca^2+^ (Ca_*V*_) channels (Huston et al., 1995). The intermolecular association underlying GPCR-mediated presynaptic inhibition at cerebellar synapses is still unresolved. Our finding suggests that compartmentalization of G_αo_ is necessary to underly presynaptic inhibition at cerebellar synapses.

The preferential localization of G_αo_ along the plasma membrane of different cerebellar neurons, either on postsynaptic and presynaptic sites, favors the classical view by which heterotrimeric G proteins remain associated with the cytoplasmic surface of the plasma membrane during their cellular signaling functions (Oldham and Hamm, 2008). However, our data revealed that G_αo_ can be found at diverse subcellular locations. We reveals that around 20% of all immunoparticles were found at intracellular sites, and most of them around subsurface cisternae, which are part of the smooth ER beneath and parallel the plasma membrane of PCs (Palay and Chan-Palay, 1974) and function as a Ca^2+^ store (Satoh et al., 1990). Similar subcellular location was described for the G_α*q/11*_ subunit (Tanaka et al., 2000), and may represent either a cytoplasmic pool of the G_αo_ subunit or a translocation molecule from the plasma membrane acting as a transducer between membrane-bound receptors and intracellular effectors.

In conclusion, we report here for the first time the cellular and ultrastructural distribution of G_αo_, which exhibit distinct patterns of subcellular localization across cerebellar neurons. This is physiologically important because implies specific functions for G_αo_ in the modulation of cellular responses and signaling cascades in a cell-type dependent manner. While further efforts are necessary to confirm the exact mechanism, our data also suggests postsynaptic and presynaptic roles for G_αo_ within these neuronal types.

## Data Availability Statement

The raw data supporting the conclusions of this article will be made available by the authors, without undue reservation.

## Ethics Statement

The animal study was reviewed and approved by Comité Ético de Experimentación Animal UCLM.

## Author Contributions

All authors had full access to all data in the study and take responsibility for the integrity of the data and the accuracy of the data analysis. RL designed the project. AR-S performed the histoblot analysis and immunohistochemical techniques at the light microscopic level. AM-B, RA-R, CA, and AM-M performed the pre-embedding immunoelectron microscopy and its quantitative analysis. RL performed the post-embedding immunoelectron microscopy and its quantitative analysis. AR-S, AM-B, RA-R, CA, and RL analyzed the data. RL wrote the manuscript. All authors read and approved the final manuscript.

## Conflict of Interest

The authors declare that the research was conducted in the absence of any commercial or financial relationships that could be construed as a potential conflict of interest.

## Footnotes

1 http://neuromab.ucdavis.edu/catalog.cfm

2 https://mouse.brain-map.org/experiment/show?id=506

## References

[B1] Aguado C. Colón J. Ciruela F. Schlaudraff F. Cabañero M. J. Perry C. (2008). Cell type-specific subunit composition of G protein-gated potassium channels in the cerebellum. *J. Neurochem.* 105 497–511. 10.1111/j.1471-4159.2007.05153.x 18088366

[B2] Aguado C. Luján R. (2019). The histoblot technique: a reliable approach to analyze expression profile of proteins and to predict their molecular association bt - co-immunoprecipitation methods for brain tissue. *Neuromethods* 144 65–88. 10.1007/978-1-4939-8985-0_6

[B3] Azam S. Haque M. E. Jakaria M. Jo S.-H. Kim I.-S. Choi D.-K. (2020). G-Protein-Coupled receptors in cns: a potential therapeutic target for intervention in neurodegenerative disorders and associated cognitive deficits. *Cells* 9:506. 10.3390/cells9020506 32102186 PMC7072884

[B4] Billinton A. Upton N. Bowery N. G. (1999). GABA B receptor isoforms GBR1a and GBR1b, appear to be associated with pre- and post-synaptic elements respectively in rat and human cerebellum. *Br. J. Pharmacol.* 126 1387–1392. 10.1038/sj.bjp.0702460 10217533 PMC1565927

[B5] Bischoff S. Leonhard S. Reymann N. Schuler V. Shigemoto R. Kaupmann K. (1999). Spatial distribution of GABA(B)R1 receptor mRNA and binding sites in the rat brain. *J. Comp. Neurol.* 412 1–16. 10.1002/(SICI)1096-9861(19990913)412:1<1::AID-CNE1<3.0.CO;2-D 10440706

[B6] Borroto-Escuela D. O. Carlsson J. Ambrogini P. Narváez M. Wydra K. Tarakanov A. O. (2017). Understanding the role of GPCR heteroreceptor complexes in modulating the brain networks in health and disease. *Front. Cell. Neurosci.* 11:37. 10.3389/fncel.2017.00037 28270751 PMC5318393

[B7] Brown D. A. Sihra T. S. (2008). Presynaptic signaling by heterotrimeric G-Proteins. *Handb. Exp. Pharmacol.* 184 207–260. 10.1007/978-3-540-74805-2_8 18064416

[B8] Cha H. L. Choi J.-M. Oh H.-H. Bashyal N. Kim S.-S. Birnbaumer L. (2019). Deletion of the α subunit of the heterotrimeric Go protein impairs cerebellar cortical development in mice. *Mol. Brain* 12:57. 10.1186/s13041-019-0477-9 31221179 PMC6585000

[B9] Choi J.-M. Kim S.-S. Choi C.-I. Cha H. L. Oh H.-H. Ghil S. (2016). Development of the main olfactory system and main olfactory epithelium-dependent male mating behavior are altered in G o -deficient mice. *Proc. Natl. Acad. Sci. U.S.A.* 113 10974–10979. 10.1073/pnas.1613026113 27625425 PMC5047177

[B10] Ciruela F. Fernández-Dueñas V. Sahlholm K. Fernández-Alacid L. Nicolau J. C. Watanabe M. (2010). Evidence for oligomerization between GABAB receptors and GIRK channels containing the GIRK1 and GIRK3 subunits. *Eur. J. Neurosci.* 32 1265–1277. 10.1111/j.1460-9568.2010.07356.x 20846323

[B11] Clancy S. M. Fowler C. E. Finley M. Suen K. F. Arrabit C. Berton F. (2005). Pertussis-toxin-sensitive Gα subunits selectively bind to C-terminal domain of neuronal GIRK channels: evidence for a heterotrimeric G-protein-channel complex. *Mol. Cell. Neurosci.* 28 375–389. 10.1016/j.mcn.2004.10.009 15691717

[B12] de Oliveira P. G. Ramos M. L. S. Amaro A. J. Dias R. A. Vieira S. I. (2019). Gi/o-protein coupled receptors in the aging brain. *Front. Aging Neurosci.* 11:89. 10.3389/fnagi.2019.00089 31105551 PMC6492497

[B13] Fajardo-Serrano A. Wydeven N. Young D. Watanabe M. Shigemoto R. Martemyanov K. A. (2013). Association of Rgs7/Gβ5 complexes with girk channels and GABA B receptors in hippocampal CA1 pyramidal neurons. *Hippocampus* 23 1231–1245. 10.1002/hipo.22161 23804514 PMC4060866

[B14] Fernández E. Collins M. O. Uren R. T. Kopanitsa M. V. Komiyama N. H. Croning M. D. R. (2009). Targeted tandem affinity purification of PSD-95 recovers core postsynaptic complexes and schizophrenia susceptibility proteins. *Mol. Syst. Biol.* 5:269. 10.1038/msb.2009.27 19455133 PMC2694677

[B15] Fernández-Alacid L. Aguado C. Ciruela F. Martín R. Colón J. Cabañero M. J. (2009). Subcellular compartment-specific molecular diversity of pre- and post-synaptic GABA B -activated GIRK channels in Purkinje cells. *J. Neurochem.* 110 1363–1376. 10.1111/j.1471-4159.2009.06229.x 19558451 PMC2774143

[B16] Fowler C. E. Aryal P. Suen K. F. Slesinger P. A. (2007). Evidence for association of GABA B receptors with Kir3 channels and regulators of G protein signalling (RGS4) proteins. *J. Physiol.* 580 51–65. 10.1113/jphysiol.2006.123216 17185339 PMC2075413

[B17] Fritschy J.-M. Weinmann O. Wenzel A. Benke D. (1998). Synapse-specific localization of NMDA and GABAA receptor subunits revealed by antigen-retrieval immunohistochemistry. *J. Comp. Neurol.* 390 194–210. 10.1002/(SICI)1096-9861(19980112)390:2<194::AID-CNE3<3.0.CO;2-X 9453664

[B18] Huston E. Cullen G. P. Burley J. R. Dolphin A. C. (1995). The involvement of multiple calcium channel sub-types in glutamate release from cerebellar granule cells and its modulation by GABAB receptor activation. *Neuroscience* 68 465–478. 10.1016/0306-4522(95)00172-F 7477957

[B19] Ige A. O. Bolam J. P. Billinton A. White J. H. Marshall F. H. Emson P. C. (2000). Cellular and sub-cellular localisation of GABA(B1) and GABA(B2) receptor proteins in the rat cerebellum. *Mol. Brain Res.* 83 72–80. 10.1016/S0169-328X(00)00199-6 11072097

[B20] Ito M. (2001). Cerebellar long-term depression: characterization, signal transduction, and functional roles. *Physiol. Rev.* 81 1143–1195. 10.1152/physrev.2001.81.3.1143 11427694

[B21] Ito M. (2006). Cerebellar circuitry as a neuronal machine. *Prog. Neurobiol.* 78 272–303. 10.1016/j.pneurobio.2006.02.006 16759785

[B22] Jiang M. Gold M. S. Boulay G. Spicher K. Peyton M. Brabet P. (1998). Multiple neurological abnormalities in mice deficient in the G protein Go. *Proc. Natl. Acad. Sci. U.S.A.* 95 3269–3274. 10.1073/pnas.95.6.3269 9501252 PMC19731

[B23] Kulik A. Nakadate K. Nyíri G. Notomi T. Malitschek B. Bettler B. (2002). Distinct localization of GABA B receptors relative to synaptic sites in the rat cerebellum and ventrobasal thalamus. *Eur. J. Neurosci.* 15 291–307. 10.1046/j.0953-816x.2001.01855.x 11849296

[B24] Laviv T. Vertkin I. Berdichevsky Y. Fogel H. Riven I. Bettler B. (2011). Compartmentalization of the GABAB receptor signaling complex is required for presynaptic inhibition at hippocampal synapses. *J. Neurosci.* 31 12523–12532. 10.1523/JNEUROSCI.1527-11.2011 21880914 PMC6703276

[B25] Li J. Ge Y. Huang J.-X. Strømgaard K. Zhang X. Xiong X.-F. (2020). Heterotrimeric G proteins as therapeutic targets in drug discovery. *J. Med. Chem.* 63 5013–5030. 10.1021/acs.jmedchem.9b01452 31841625

[B26] Lin M. T. Luján R. Watanabe M. Adelman J. P. Maylie J. (2008). SK2 channel plasticity contributes to LTP at Schaffer collateral–CA1 synapses. *Nat. Neurosci.* 11 170–177. 10.1038/nn2041 18204442 PMC2613806

[B27] Luján R. Nusser Z. Roberts J. D. B. Shigemoto R. Somogyi P. (1996). Perisynaptic location of metabotropic glutamate receptors mGluR1 and mGluR5 on dendrites and dendritic spines in the rat hippocampus. *Eur. J. Neurosci.* 8 1488–1500. 10.1111/j.1460-9568.1996.tb01611.x 8758956

[B28] Martín-Belmonte A. Aguado C. Alfaro-Ruíz R. Moreno-Martínez A. E. de la Ossa L. Martínez-Hernández J. (2020). Reduction in the neuronal surface of post and presynaptic GABAB receptors in the hippocampus in a mouse model of Alzheimer’s disease. *Brain Pathol.* 30 554–575. 10.1111/bpa.12802 31729777 PMC7317930

[B29] Neer E. J. (1995). Heterotrimeric C proteins: organizers of transmembrane signals. *Cell* 80 249–257. 10.1016/0092-8674(95)90407-7 7834744

[B30] Oldham W. M. Hamm H. E. (2008). Heterotrimeric G protein activation by G-protein-coupled receptors. *Nat. Rev. Mol. Cell Biol.* 9 60–71. 10.1038/nrm2299 18043707

[B31] Palay S. L. Chan-Palay V. (1974). *Cerebellar Cortex.* Berlin: Springer-Verlag.

[B32] Rhodes K. J. Trimmer J. S. (2008). Antibody-based validation of CNS ion channel drug targets. *J. Gen. Physiol.* 131 407–413. 10.1085/jgp.200709926 18411328 PMC2346570

[B33] Satoh T. Ross C. A. Villa A. Supattapone S. Pozzan T. Snyder S. H. (1990). The inositol 1,4,5,-trisphosphate receptor in cerebellar Purkinje cells: quantitative immunogold labeling reveals concentration in an ER subcompartment. *J. Cell Biol.* 111 615–624. 10.1083/jcb.111.2.615 2166053 PMC2116203

[B34] Schüller U. Lamp E. C. Schilling K. (2001). Developmental expression of heterotrimeric G-proteins in the murine cerebellar cortex. *Histochem. Cell Biol.* 116 149–159. 10.1007/s004180100303 11685543

[B35] Schwenk J. Pérez-Garci E. Schneider A. Kollewe A. Gauthier-Kemper A. Fritzius T. (2016). Modular composition and dynamics of native GABAB receptors identified by high-resolution proteomics. *Nat. Neurosci.* 19 233–242. 10.1038/nn.4198 26691831

[B36] Simon M. Strathmann M. Gautam N. (1991). Diversity of G proteins in signal transduction. *Science (80-.).* 252 802–808. 10.1126/science.1902986 1902986

[B37] Tanaka J. Nakagawa S. Kushiya E. Yamasaki M. Fukaya M. Iwanaga T. (2000). Gq protein α subunits Gαq and Gα11 are localized at postsynaptic extra-junctional membrane of cerebellar Purkinje cells and hippocampal pyramidal cells. *Eur. J. Neurosci.* 12 781–792. 10.1046/j.1460-9568.2000.00959.x 10762307

[B38] Watanabe M. Fukaya M. Sakimura K. Manabe T. Mishina M. Inoue Y. (1998). Selective scarcity of NMDA receptor channel subunits in the stratum lucidum (mossy fibre-recipient layer) of the mouse hippocampal CA3 subfield. *Eur. J. Neurosci.* 10 478–487. 10.1046/j.1460-9568.1998.00063.x 9749710

[B39] Weis W. I. Kobilka B. K. (2018). The molecular basis of G protein–coupled receptor activation. *Annu. Rev. Biochem.* 87 897–919. 10.1146/annurev-biochem-060614-033910 29925258 PMC6535337

[B40] Wettschureck N. Offermanns S. (2005). Mammalian G proteins and their cell type specific functions. *Physiol. Rev.* 85 1159–1204. 10.1152/physrev.00003.2005 16183910

[B41] Worley P. F. Baraban J. M. Van Dop C. Neer E. J. Snyder S. H. (1986). Go, a guanine nucleotide-binding protein: immunohistochemical localization in rat brain resembles distribution of second messenger systems. *Proc. Natl. Acad. Sci. U.S.A.* 83 4561–4565. 10.1073/pnas.83.12.4561 3086888 PMC323774

