# Supplementary material for: Cellular Diversity and Differential Subcellular Localization of the G-Protein Gαo Subunit in the Mouse Cerebellum
Source: Front Neuroanat. 2021 Jun 25;15:686279. doi: 10.3389/fnana.2021.686279 (PMC8267243; doi:10.3389/fnana.2021.686279)
Supplement: Supplementary file 1 [file Data_Sheet_1.PDF]

Cellular diversity and differential subcellular localisation of the G-protein $G_{\alpha o}$ subunit in the mouse cerebellum

Alberto Roldán-Sastre[1], Carolina Aguado[1], Alejandro Martín-Belmonte[1], Rocío Alfaro-Ruiz[1] Ana Esther Moreno-Martínez[1] and Rafael Luján[1]

[1]*Synaptic Structure Laboratory*, Instituto de Investigación en Discapacidades Neurológicas (IDINE), Dept. Ciencias Médicas, Facultad de Medicina, Universidad Castilla-La Mancha, Campus Biosanitario, C/ Almansa 14, 02008 Albacete, Spain.

SUPPLEMENTARY INFORMATION

Antibodies

We used two antibodies recognising distinct epitopes of the $G_{\alpha o}$ protein and compared the labelling patterns of the two antibodies using immunoelectron microscopy.

The following two primary antibodies against $G_{\alpha o}$ were used:

1) rabbit anti-$G_{\alpha o}$ polyclonal (ref#ab154001; Recombinant fragment corresponding to Human GNAO1 aa 104-338; Abcam, Cambridge, UK);

2) monoclonal anti-$G_{\alpha o}$ (MAB3073; clone 2A; raised against Gαo of bovine origin, Millipore Corporation, Burlington, MA, USA). We have provided information about the specificity of $G_{\alpha o}$ antibodies in the cerebellum (see below).

When immunohistochemistry at the light microscopic level was used, we observed virtually the same staining pattern in the cerebellar cortex for the two antibodies (**Supplementary Figure 1**). Similarly, when pre-embedding immunogold approaches were applied, we obtained virtually the same subcellular localisation pattern in the same cell types and neuronal compartments for the two antibodies

(**Supplementary Figure 2**), though the polyclonal antibody raised in rabbit provided a stronger labelling pattern than the monoclonal antibody. This concordant data using two different technical approaches indicates that labelling pattern represents a specific signal.

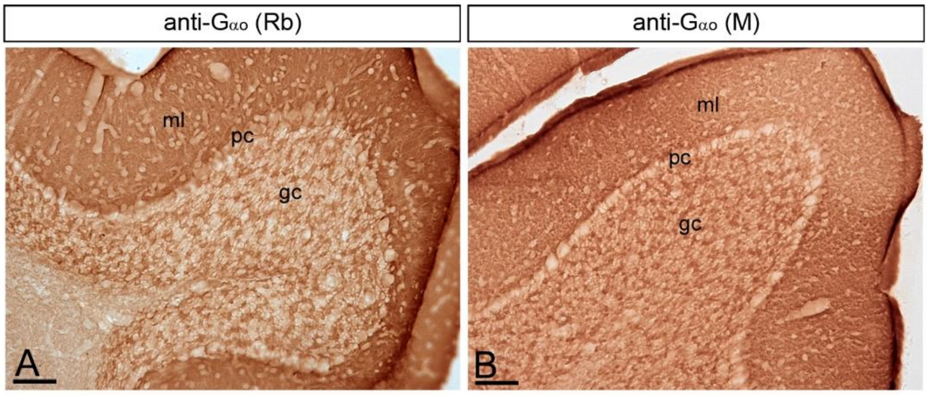

Supplementary Figure 1. Immunoreactivity for $G_{\alpha o}$ in the cerebellar cortex of as revealed at the light microscopy level using two different antibodies against $G_{\alpha o}$. Staining for $G_{\alpha o}$ in the cerebellum using the rabbit antibody (*panel A*) and the mouse antibody (*panel B*), processed in parallel, showed the same labelling pattern. Scale bars: 10 µm.

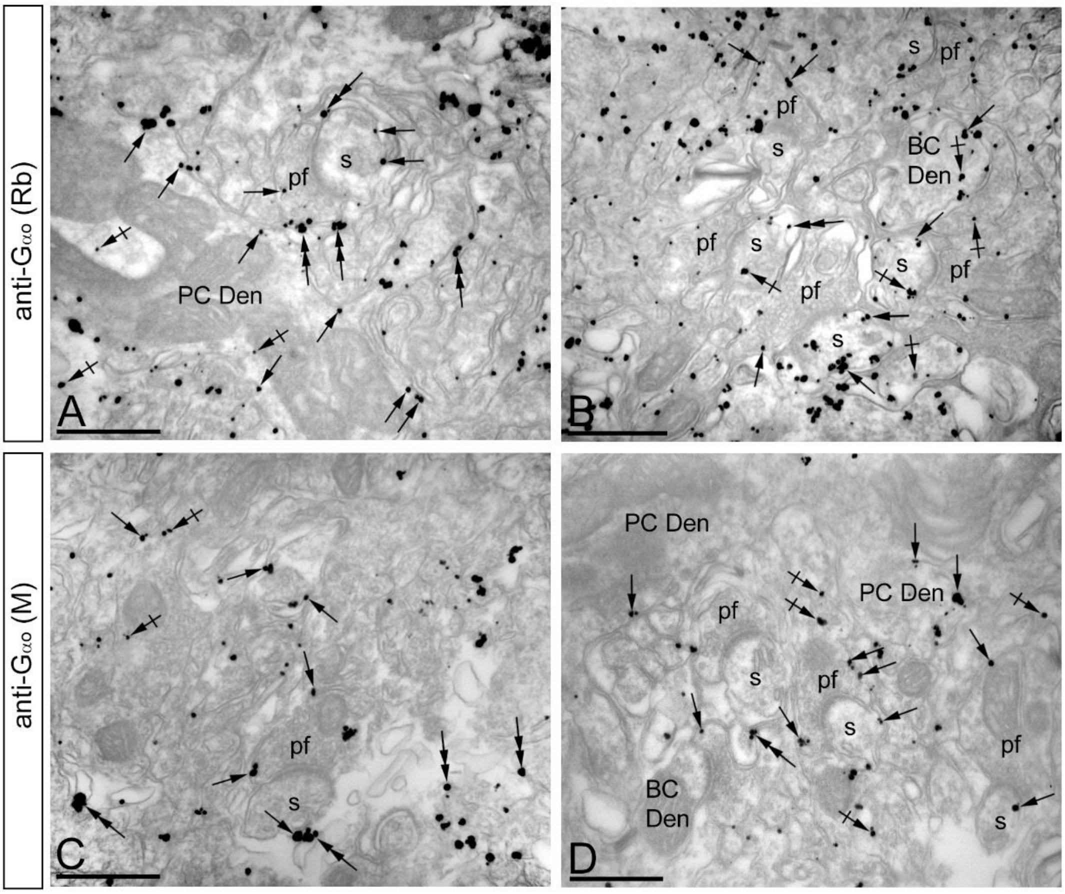

Supplementary Figure 2. ***Specificity of antibodies against $G_{\alpha o}$ in the cerebellar cortex using immunoelectron microscopy.*** Electron micrographs showing immunoparticles for $G_{\alpha o}$ in the molecular layer of the cerebellum, as detected using a pre-embedding immunogold technique. (A,B) Using the antibody raised in rabbit, immunoparticles for $G_{\alpha o}$ were distributed along the extrasynaptic plasma membrane (arrows) of dendritic shafts (PC Den) and spines (s) of PCs contacted by parallel fibre terminals (pf). To a lesser extent, immunoparticles for $G_{\alpha o}$ were detected at intracellular sites (crossed arrows) associated with intracellular membranes. Presynaptically, $G_{\alpha o}$ immunoparticles were also distributed along the plasma membrane and at intracellular sites in axon terminals of parallel fibres (pf). In addition to PCs, immunoparticles for $G_{\alpha o}$ were detected in Bergmann glia cells (double arrows). (C,D) Using the monoclonal antibody, immunoparticles for $G_{\alpha o}$ were distributed in the same compartment showing the same subcellular localisation pattern, although with lower efficiency than the polyclonal antibody. BC, basket cell dendrite. Scale bars: A-D, 500 nm.
